# Impact of Medication Regimen Simplification on Medication Incidents in Residential Aged Care: SIMPLER Randomized Controlled Trial

**DOI:** 10.3390/jcm10051104

**Published:** 2021-03-06

**Authors:** Nicolas Dugré, J. Simon Bell, Ria E. Hopkins, Jenni Ilomäki, Esa Y. H. Chen, Megan Corlis, Jan Van Emden, Michelle Hogan, Janet K. Sluggett

**Affiliations:** 1 Centre for Medicine Use and Safety, Faculty of Pharmacy and Pharmaceutical Sciences, Monash University, Melbourne, VIC 3052, Australia; dugre.nicolas@gmail.com (N.D.); ria.hopkins@monash.edu (R.E.H.); jenni.ilomaki@monash.edu (J.I.); esa.chen@monash.edu (E.Y.H.C.); janet.sluggett@unisa.edu.au (J.K.S.); 2 Faculty of Pharmacy, Université de Montréal, Montréal, QC H3C 3J7, Canada; 3 Centre Intégré Universitaire de Santé et de Services Sociaux du Nord-de-l’Île-de-Montréal, Clinique Universitaire de Médecine Familiale Sacré-Coeur, Montréal, QC H3M 3A9, Canada; 4 NHMRC Cognitive Decline Partnership Centre, Hornsby Ku-ring-gai Hospital, Sydney, NSW 2077, Australia; mcorlis@helpinghand.org.au (M.C.); JVanEmden@helpinghand.org.au (J.V.E.); mehogan@helpinghand.org.au (M.H.); 5 Department of Epidemiology and Preventative Medicine, Monash University, Melbourne, VIC 3004, Australia; 6 Helping Hand Aged Care, Adelaide, SA 5006, Australia; 7 UniSA Allied Health and Human Performance Unit, City East campus, University of South Australia, Adelaide, SA 5001, Australia

**Keywords:** long-term care, medication administration errors, medication incidents, medication regimen simplification, nursing homes, residential aged care

## Abstract

In the SImplification of Medications Prescribed to Long-tErm care Residents (SIMPLER) cluster-randomized controlled trial, we investigated the impact of a structured medication regimen simplification intervention on medication incidents in residential aged care facilities (RACFs) over a 12-month follow-up. A clinical pharmacist applied the validated 5-step Medication Regimen Simplification Guide for Residential Aged CarE (MRS GRACE) for 96 of the 99 participating residents in the four intervention RACFs. The 143 participating residents in the comparison RACFs received usual care. Over 12 months, medication incident rates were 95 and 66 per 100 resident-years in the intervention and comparison groups, respectively (adjusted incident rate ratio (IRR) 1.13; 95% confidence interval (CI) 0.53–2.38). The 12-month pre/post incident rate almost halved among participants in the intervention group (adjusted IRR 0.56; 95%CI 0.38–0.80). A significant reduction in 12-month pre/post incident rate was also observed in the comparison group (adjusted IRR 0.67, 95%CI 0.50–0.90). Medication incidents over 12 months were often minor in severity. Declines in 12-month pre/post incident rates were observed in both study arms; however, rates were not significantly different among residents who received and did not receive a one-off structured medication regimen simplification intervention.

## 1. Introduction

Medication errors are estimated to cost USD 42 billion annually, or 0.7% of global health expenditure [1]. Medication Without Harm is the World Health Organization’s (WHO) Third Global Patient Safety Challenge, and Medication Safety was recently declared an Australian national health priority area [2,3]. Medication errors and incidents have been defined as “any preventable event that may cause or lead to inappropriate medication use or patient harm while the medication is in the control of the health care professional, patient, or consumer” [4]. Incidents can arise at points in the medication management cycle including prescribing, dispensing, administration and monitoring [5,6]. A review of 36 studies across all United Kingdom (UK) National Health Service (NHS) settings reported medication error rates from 0.2% (prescribing error rate at hospital discharge) to 90.6% (proportion of residents of aged care facilities who received a potentially inappropriate medication) [7], while a systematic review of 91 direct observation studies of the NHS reported a median error rate including dose timing errors of 19.6% [8].

There is a high potential for medication incidents in residential aged care facilities (RACFs) due to high rates of multimorbidity, polypharmacy, and frequent transitions of care [9,10,11,12]. Medications often implicated in errors, such as psychotropic medications, opioids, anticoagulants, antidiabetic agents and diuretics are prevalent in RACFs [9,11,12]. A UK care home study reported four-fold higher incident rates for liquid medications and 19-fold higher for topical, injectable, or transdermal medications compared to tablets and capsules [13]. In Australia, medication management is the leading source of complaints regarding residential aged care [14]. A systematic review of medication errors in RACFs reported 16–27% of residents experienced a medication error, with 13–31% of hospital transfers examined in three studies due to medication errors [15]. One UK study involving interviews, case note review, direct observation and inspection of dispensing records reported that errors occurred in 70% of residents, while a second UK study determined 90% of residents had one or more administration errors over a three-month period [16,17]. Underreporting of errors is variable and may be due to inaccessible or difficult reporting systems, limited understanding of reporting, and fear of punitive action [11,15,18]. Apparent variability in error rates may also be explained by different methods for ascertaining and categorizing errors.

Interventions to reduce incidents include electronic or standardized medication administration charts, medication adherence aids, medication distribution technologies, computerized decision support and embedding pharmacists within RACFs [19,20,21,22,23]. No randomized controlled trial (RCT) has evaluated the impact of simplifying medication regimens on medication incidents in RACFs. Medication regimen complexity can arise due to number of medications, multiple administration times, non-oral formulations, and additional dosing instructions (e.g., crush tablets, administer with food) [24,25]. Residents with more complex medication regimens are more likely to be hospitalized over a 12-month period [26]. In hospital settings, number of medication doses and unscheduled dosing times are associated with medication incidents [27].

The SImplification of Medications Prescribed to Long-tErm care Residents (SIMPLER) study is a three-year cluster randomized controlled trial involving 242 participants [28]. The overall objective of the SIMPLER study was to improve resident health and quality of life through reducing the number of daily medication administration times. Medication simplification was possible for 62 (65%) of the 99 residents in the intervention arm of the SIMPLER study and 57 (62%) of 92 simplification recommendations made by the pharmacist delivering the intervention were implemented by four- month follow-up. The most frequent recommendations were to change an administration time (65%), formulation (27%), or dose frequency (4%). At four-month follow-up the mean number of medication administration times (the primary outcome) was significantly reduced in the intervention compared to comparison arm (−0.36, 95% confidence intervals (CI) −0.63 to −0.09, *p* = 0.01) and this was maintained at eight- and 12-month follow-up [29,30]. Although the rate of medication incidents was greater in the intervention arm compared to the comparison arm at four-month follow-up in the unadjusted analyses (incident rate ratio (IRR) 1.91, 95% CI 1.02 to 3.67), no significant difference was observed after adjustment for the rate of medication incidents in the four months pre-study entry (IRR 1.55, 95% CI 0.81 to 2.91, *p* = 0.17). The objective of this planned secondary outcome analysis was to investigate the impact of medication regimen simplification on medication incidents at 12-month follow-up of the SIMPLER study.

## 2. Experimental Section

### 2.1. Study Design

The SIMPLER study is an open-label, matched-paired cluster randomized controlled trial involving eight RACFs [28]. Participating residents from the four RACFs randomized to the intervention arm received a one-off clinical pharmacist simplification intervention. Residents of the four comparison RACFs received usual care. In Australia, medications are prescribed and dispensed by off-site physicians and pharmacists and administered by RACF staff to residents who are often living with cognitive impairment or dementia [11,12]. The eight participating RACFs used hard copy medication charts, with medications administered to residents from pre-packed dose administration aids (e.g., blister packs, sachets). The study was approved by the Monash University Human Research Ethics Committee (0781) and the participating aged care provider organization. Written informed consent was obtained from participants or from their guardian, next of kin, or significant other when the resident was unable to provide written informed consent to participate. The SIMPLER trial was registered with the Australian New Zealand Clinical Trials Registry (ACTRN12617001060336).

### 2.2. Participants

Participants were recruited between April and October 2017. All English-speaking residents taking at least one regular medication were eligible. Residents were excluded if RACF staff deemed they were medically unwell or were estimated to have less than three months to live. The 242 participating residents were similar to all residents of Australian RACFs in terms of age (62% vs. 59% aged 85 years or older), sex (74% vs. 67% female), and length of RACF stay (2.5 years vs. 2.9 years) [31].

### 2.3. Intervention

The intervention was a one-off application of the Medication Regimen Simplification Guide for Residential Aged CarE (MRS GRACE) [32]. MRS GRACE is a structured, validated implicit tool to assist pharmacists and other clinicians to identify opportunities for medication simplification. An experienced clinical pharmacist reviewed medication charts for participants in the four intervention RACFs and used the principles outlined in the MRS GRACE to identify opportunities to simplify regular medications. Regimen simplification involved consolidating administration times through administering medications at the same time, standardizing routes of administration, using long-acting rather than short-acting formulations, and switching to combination rather than single-ingredient formulations, where possible [28]. The most common recommendations made involved adjusting the timing of medication dosing: for example, consolidating medications taken at 07:00 am and 08:00 am, if appropriate. Other common recommendations included changing paracetamol from immediate-release tablets prescribed four times daily, to sustained-release tablets prescribed three times daily, and using combination products (e.g., metformin 1000 mg tablet and saxagliptin 5 mg tablet was changed to saxagliptin/metformin 5 mg/1000 mg tablet) [29].

Recommendations arising from the intervention were communicated to the residential services manager (RSM) or clinical nurse consultant at the RACF and general practitioner (GP), who were responsible for reviewing and implementing the simplification recommendations.

### 2.4. Outcomes

In this planned secondary analysis, the outcome of interest was the number of medication incidents in the 12 months following the intervention. Medication incident data were extracted from the organization’s risk management and reporting system, which was uniform across the eight RACFs. Incidents were entered into the database after detection by the RACF staff according to the organization’s Client Incident Reporting Policy. The client incident reports capture information using a combination of radio buttons and free text fields, including the incident date, time, personnel involved, person completing the form, specific location of the incident, description of the incident, immediate action taken, outcomes of investigations and other findings, controls/strategies implemented in response to the incident, hospital transfer details, family/police notifications, and additional information about the specific incident type. Incidents were then reviewed by the RACF RSM who classified the incident by type as an administration error (incorrect medication/dose/route, incorrect time/date, missing medication/medication not available, omission, other), adverse drug reaction, resident error, pharmacy dispensing error, or prescribing error. Incident severity and response were determined by the RSM using a Severity Assessment Code (SAC) matrix combining the impact of the incident with the likelihood of occurrence. This is a widely used approach in Australia and internationally and is consistent with the approach advocated by SA Health in South Australia where the RACFs were located [33]. RSMs had previously been trained on the use of the SAC Matrix and risk assessment processes. The SAC matrix is used to assess the severity of all incidents within the RACF (i.e., medication incidents, falls, near misses, incidents relating to client behaviour) and considers both resident, staff, and organizational consequences. First, the general impact of the incident is categorized as minimal, minor, moderate, major, or severe. Minor events include near misses and events managed with existing processes that did not result in resident injury or service disruption. Examples of severe events include resident or staff death, or complete loss of service provision. The likelihood of occurrence is then categorized as rare (i.e., unlikely to occur or may happen in 5–30 years), unlikely, possible, likely, or frequent (i.e., expected to recur either immediately or within weeks/months). The incident is then categorized using the SAC matrix to produce a final score from 1 to 4, with a lower SAC score representing an extreme risk (Supplementary Table S1). An incident with an SAC of 4 was managed through routine procedures while an incident with an SAC of 1 required immediate escalation to the chief executive officer and other executive members.

Incident data were then extracted for analysis by the research team after all participants had completed 12-months follow-up. Medications involved in incidents were classified retrospectively by researchers using the WHO Anatomical Therapeutic Chemical (ATC) classification system at the third level (therapeutic/pharmacological subgroup) [34], based on the information entered by the reporting RACF staff member.

### 2.5. Covariates

Baseline demographic data included age, gender, RACF location, and length of stay at the RACF. Medication data collected included number of charted medications, and number of regularly charted daily administration times. Comorbidity data were used to calculate Charlson Comorbidity Index [35], and frailty using the 7-item FRAIL-NH scale [36]. Medication incidents for each resident for the 12 months prior to study recruitment were also collected from the risk reporting software (12-month pre-rate). The 12-month pre-rate and any baseline demographics demonstrating significant differences between arms (*p* < 0.1) were included as covariates in analyses.

### 2.6. Analysis

Participant, incident, and medication characteristics were reported using descriptive statistics. Negative binomial regression was used to conduct intention-to-treat analysis for the associations between the intervention and medication incidents. In addition, incident rates were compared for the 12 months pre- and poststudy entry within each study arm. The results were expressed in incidents per 100 resident-years and associations were reported using IRRs with 95% CIs. This method considered that each resident contributed different lengths of follow-up time. Resident time contributed to the study was calculated taking into consideration date of entry to the RACF (pre-study entry period), date of death (post-study entry period), and days spent in hospital (both periods). RACF was included in models as a random effect to account for clustering. Two sets of per-protocol analyses were undertaken, firstly, only including residents in the intervention arm with at least one simplification recommendation and, secondly, only including residents in the intervention arm with at least one simplification recommendation implemented. We also conducted an additional sensitivity analysis by only including residents with at least two or more medication administration times at baseline. Analyses were undertaken using SAS version 9.4 (SAS Institute, Cary, NC, USA) and SPSS version 25.0 (IBM, Armonk, NY, USA), with *p* < 0.05 considered statistically significant.

## 3. Results

### 3.1. Demographics

**Figure 1:**
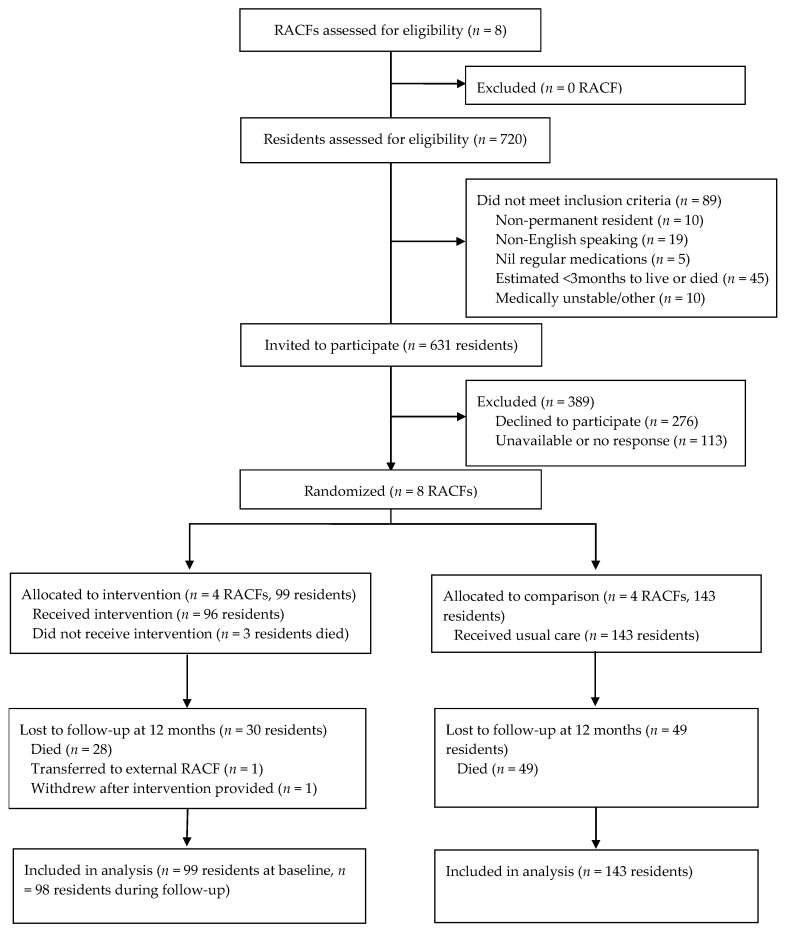
Study flow diagram. RACF: Residential aged care facility.

There were 99 residents in the four intervention RACFs and 143 residents in the comparison RACFs. Follow-up data were available for 241 residents (Table 1): one intervention arm resident withdrew from the trial after randomization and received no simplification recommendations (Figure 1). Overall, 162 residents were alive and followed up at 12 months (intervention arm = 69; comparison arm = 93). Residents in the comparison arm were more likely to be female, live in an urban area and have a longer duration of stay. Comorbidity scores and the number of medications charted for regular administration at baseline were similar in both groups.

### 3.2. Number and Type of Medication Incidents during Follow-Up

There were 148 medication incidents reported for 31% of residents during the 12-month follow-up (Table 2). This included 72 incidents among 34 residents in the intervention arm (34%) and 76 incidents among 40 residents (28%) in the comparison group (range 0–7 per resident). Incident rates per facility ranged from 16 to 165 incidents per 100 person-years. In total, 126 medication incidents (85.1%) were medication administration incidents. A severity score was assigned for 145 incidents, of which 137 (94.5%) had an SAC of 4, and eight (5.5%) received an SAC of 3 (Supplementary Table S1). No medication incidents resulted in hospitalization.

The specific medications involved in the incident were documented for 76 of the 148 incidents. The most commonly implicated medications according to ATC code were antithrombotic agents (*n* = 11) and other analgesics, namely paracetamol (acetaminophen) (*n* = 11) (Supplementary Table S2). The incident was attributed to a Drug of Dependence or Addiction (DDA) for 54 incidents (36.4%) however the specific agent involved was not named. Incidents most commonly involved oral medications (55.4%) and transdermal (35.8%) preparations. Almost all incidents involved regularly administered medications (89.9%).

### 3.3. Medication Incident Rates

**Figure 2:**
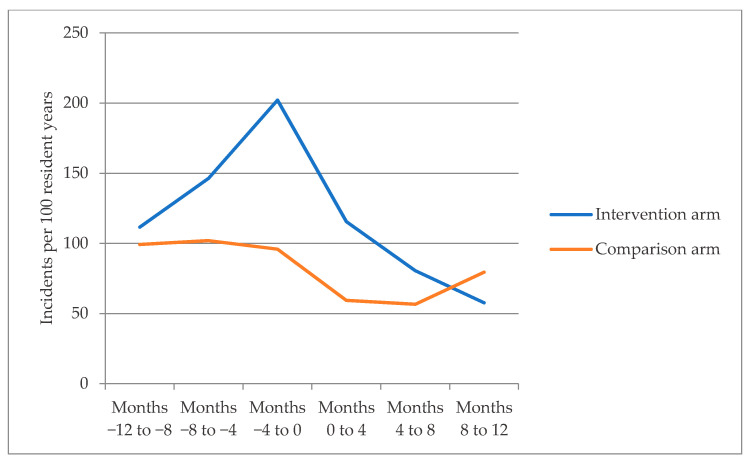
Medication incident rates in the intervention and comparison residential aged care facilities.

**Figure 3:**
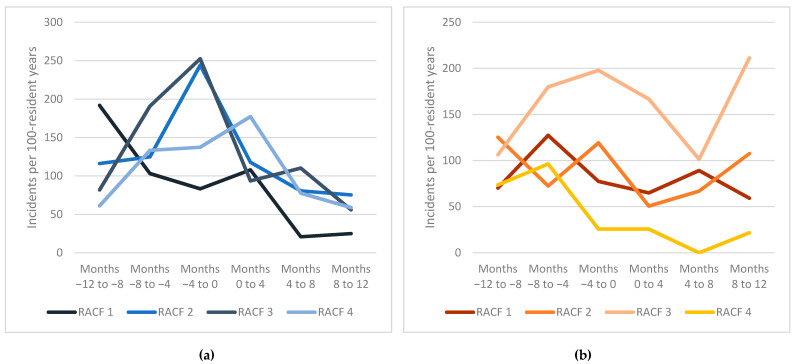
Medication incident rates in individual intervention (**a**) and comparison (**b**) residential aged care facilities. RACF: Residential aged care facility.

Over 12 months, mean medication incident rates were 95 and 66 per 100 patient-years in the intervention group and the comparison group, respectively (adjusted IRR 1.13; 95% CI 0.53–2.38) (Table 3). During the 12 months preceding the study, residents in the intervention group had more medication incidents than residents in the comparison arm (161 vs. 97 incidents per 100 person-years, IRR 1.65; 95%CI 1.18–2.31). In the intervention group, the medication incident rate was significantly reduced during 12-month follow-up in comparison to the rate observed in the 12-months before study entry (95 incidents per 100 person-years vs. 161 incidents per 100 person-years, IRR 0.56; 95% CI 0.38–0.80). In the comparison arm, there was a nearly one-third reduction in medication incidents (64 incidents per 100 person-years vs. 97 incidents per 100 person-years, IRR 0.67, 95% CI 0.50–0.90) (Figure 2). Incident rates for individual facilities is presented in Figure 3.

### 3.4. Per Protocol Analysis

There were no significant differences between the intervention and comparison groups when only intervention participants with at least one recommendation (*n* = 62) were included (adjusted IRR 1.20; 95% CI 0.55–2.63), or intervention participants with at least one implemented recommendation (*n* = 46) were included (adjusted IRR 1.08; 95% CI 0.43–2.70). In sensitivity analyses only including residents with at least two daily administration times (*n* = 235), no significant differences between study arms were observed (adjusted IRR 1.09, 95% CI 0.61–1.95).

## 4. Discussion

The SIMPLER study is the first RCT to investigate the impact of medication regimen simplification on medication incidents in RACFs. A decline in medication incidents over time was observed in both the intervention and comparison arms. However, medication incident rates were not significantly different among residents in the intervention and comparison arm over 12 months of follow-up.

There are a number of mechanisms that may explain the decline in incidents. Our results were consistent with less complex medication regimens being associated with lower incident rates [27]. Although not statistically significant, there was a 30% lower incident rate in favour of the intervention group after eight months of follow-up. While there was considerable facility-to-facility variability in incident reports in both intervention and comparison RACFs, there was a downward trend across all four intervention RACFs. The lack of significance may be attributable to insufficient statistical power due to a limited number of clusters, participants, and incidents. We believe the decline in medication incidents in both arms was unlikely to be attributable to the Hawthorne effect arising from nurses being aware of the SIMPLER trial, as it is unlikely that nurses responsible for medication administration would recall which residents participated in the trial and adjust their behaviour over a 12-month period. This is supported by previous research reporting limited evidence for the Hawthorne effect in health professional education research [37]. All intervention and comparison RACFs had a uniform Client Incident Reporting Policy, however, facility-to-facility variation may have arisen due to the complex nature of medication incident reporting [11].

A previous study of embedding a pharmacist within a RACF for six months in Canberra, Australia resulted in an apparent increase in medication incidents [23]. This may be because the pharmacist increased detection and reporting of incidents, either directly themselves or by nurses and GPs involved in the medication review process. However, we observed a decline rather than increase in medication incidents in both the intervention and comparison arms of the SIMPLER study. We had anticipated a small increase in incidents may have occurred immediately after a medication regimen simplification intervention due to changes to dose times and formulations. However, we did not find any evidence for this.

Approximately one-third of study participants experienced a medication incident, which is slightly higher than the 16–27% of residents in a systematic review of 11 studies [11]. However, by extrapolating based on the average number of daily medication administration times and total resident-days of follow-up, we estimate that the 148 medication incidents in our study translates to an error in less than 0.1% of medication administrations. Other studies have reported considerably higher rates of medication incidents: two-thirds of participants in Barber’s study experienced an error [16], while Szcepura et. al. reported 90% of residents were exposed to medication administration errors over a three-month observation period [17]. However, these studies identified errors prospectively rather than through routine reporting. Barber et al. also reported 39% of residents had prescribing errors and 22% had administration errors [16]. In our study, the majority of incidents were administration errors; there were no prescribing errors and few dispensing errors reported. This finding likely reflects that incidents were predominately reported by nurses who were responsible for medication administration rather than prescribing. In our study, medication incidents were assessed by nursing staff to be of low-moderate severity, with no incidents scoring “extreme” or “high” SAC codes. This is in line with most medication incidents reported in other studies not having been associated with major adverse events [7,15].

The most frequently implicated medications were those affecting the central nervous system, alimentary tract and metabolism, cardiovascular system, and blood and blood forming organs. This finding is similar to previous studies. In a cross-sectional study of medication incidents in US RACFs, Desai et al. reported that analgesics and anxiolytics were implicated in 20% of incidents, followed by antidiabetics and anticoagulants [38]. In a systematic review of 91 studies across healthcare settings, common medications implicated in incidents included nervous system, gastrointestinal, blood and cardiovascular system, and anti-infective agents [8]. Over one third of incidents involved a DDA administration which must be overseen and documented by two staff members; having a second staff member oversee DAA administration may reduce the risk of resident harm but increase the likelihood of error detection and reporting. Over half (55%) of incidents in our study involved oral medications, however, oral medications comprised 75% of all regularly administered medications [39]. Transdermal formulations accounted for 36% of all incidents, which was consistent with research suggesting errors with transdermal administration are common and can occur at all stages including preparation, application, removal, monitoring and disposal [40]. Lampert et al. suggested a lack of knowledge and awareness regarding correct administration procedures is a root cause of medication incidents related to transdermal administration [40]. The likelihood of error may be increased because not all transdermal formulations have a consistent dosing interval.

A time-and-motion study conducted in conjunction with the SIMPLER randomized controlled trial found nurses take an average of 5 min per resident per round to administer medications [41]. Neither the time-and-motion study nor the present study investigated the time needed to safely administer different dose forms. However, we have estimated by extrapolating the reduction in average number of administration times at the 4-month follow up across a 100 bed RACF, the intervention would generate savings of 85 h of staff time per month [30]. This represents time that could be directed to other care, quality, and safety related activities. This includes implementing enhanced medication management activities. Although regimen simplification was not associated with a significant reduction in medication incidents in the intervention compared to the comparison group, complex medication regimens are burdensome for residents and staff. For this reason, medication regimen simplification remains a potentially important and worthwhile activity in the RACF setting.

### Strengths and Limitations

Our trial has several strengths. It was the first RCT on this topic. We used a matched pair cluster randomized design to avoid potential contamination associated with the same nurses and GPs providing care to residents in the intervention and comparison arms. The simplification intervention was resident-centered and consistent with Australia’s Aged Care Quality Standards that recognize that residents are important contributors in decisions about care they receive. Participants were followed over 12-months with no unexplained loss to follow-up. Incident rates were calculated in terms of person-years to account for varying lengths of follow-up. The intervention was implemented using a validated tool developed by a multidisciplinary team [32]. Incidents were also reported in both arms using the same standardized risk reporting system.

Our study also has several limitations. Our data likely represent an underestimate of the true numbers of medication incidents due to underreporting, which is a known issue with retrospectively evaluating incidents. No prescribing incidents or adverse drug reactions were reported in our study. This is likely to reflect a system-level reporting issue rather than the absence of these incidents in practice. Incident reporting systems for care organizations differ between and within countries. In Australia there is no national standard reporting system for medication incidents. Instead, aged care provider organizations develop and follow their own policies, with guidance provided by accrediting bodies regarding the recording and reporting of incidents. The Guiding Principles for Medication Management in Residential Aged Care (2012) published by the Australian Government Department of Health and Ageing also briefly outlines each aged care provider organizations’ responsibility in terms of incident and error reporting [42]. Medication incidents are typically tabled and discussed at each aged care provider organization’s multidisciplinary medication advisory committees (MACs) [43]. Medication incident reporting was by RACF staff as part of routine care rather than trained study personnel. Furthermore, due to the multi-site nature of the SIMPLER RCT, there were multiple RACF staff involved in assigning an SAC for each incident which may have contributed to intra-facility variation in reporting. The participating RACFs used hard copy medication charts. Research conducted in the hospital setting suggests different types of medication incidents may occur when electronic medication management systems are used instead of paper-based medication management systems [44]. This research identified that introduction of electronic prescribing and administration systems was associated with an increase in specific errors (e.g., wrong route, wrong formulation) but mitigation of other errors (e.g., wrong dose due to poor handwriting) [44]. Electronic charts may also be more difficult to edit and annotate than paper-based charts, with possible discrepancies due to delay or failure to update the electronic medication administration chart after a paper-based prescription is issued [45]. This may mean our findings are not fully generalizable to settings in which electronic medication management systems are used. Similarly, our findings are not generalizable to recipients of community-based home care services where medication administration is not typically undertaken by nurses. However, we have piloted a similar medication simplification intervention among recipients of community-based home care services [46]. Due to the cluster randomized design, there were different numbers of participating residents in the intervention and comparison arms and several baseline differences between arms. We adjusted our analyses for these baseline differences where possible. Participants in the intervention arm had shorter duration of stay in RACFs, though the median was still over two years. It is possible that more recently admitted residents were more prone to medication changes and, therefore, to medication related incidents. There could have been unmeasured differences between intervention and comparison RACFs regarding nursing (e.g., experience, nursing time, reporting rates) or management practices. In addition, over 35% of “administration incidents” were not sub-categorized according to type, and free text descriptions of incidents were not collected.

## 5. Conclusions

Medication incident rates were not significantly different among residents who received and did not receive a one-off structured medication regimen simplification intervention. Although the intervention did not result in a significant reduction in incidents, the 30% lower incident rate in the intervention group after eight months suggests regimen simplification may still be worth investigating as a potential strategy to reduce incidents. Given that complex medication regimens are burdensome for residents and staff, it is possible that the benefits of simplification may extend beyond the impact on medication incident rates.

## Figures and Tables

**Table 1:** Baseline characteristics of participating residents.

Characteristic	Intervention Group (*n* = 99)	Comparison Group (*n* = 143)
Age, years (median, IQR *)	86 (80–92)	88 (81–92)
Female (*n*, %)	67 (67.7)	112 (78.3)
Urban location (*n*, %)	67 (67.7)	127 (88.8)
Length of stay in RACF ^†^, years (median, IQR)	2.3 (0.9–3.6)	3.7 (14.9)
Number of medications charted (median, IQR)	12 (9–16)	13 (10–18)
Number of daily regular medication administration times (median, IQR)	4 (3–5)	4 (3–5)
FRAIL-NH ^‡^ score (median, IQR)	6 (3–9)	7 (3–10)
Charlson Comorbidity Index score (median, IQR)	2 (1–4)	2 (1–3)

* IQR, interquartile range; ^†^ RACF, residential aged care facility; ^‡^ FRAIL-NH, 7-item Fatigue, Resistance, Ambulation, Incontinence or illness, Loss of weight, Nutritional status, and Help with dressing in nursing homes scale.

**Table 2:** Characteristics of medication incidents at 12 months.

*n* (%)	Intervention Group Incidents = 72	Comparison Group Incidents = 76
**Incident classification**		
Administration error	64 (88.8)	62 (81.5)
Wrong drug/dose/route	3 (4.1)	1 (1.3)
Wrong time/date	1 (1.3)	2 (2.6)
Medication missing or N/A	8 (11.1)	22 (28.9)
Omission	19 (26.4)	18 (23.7)
Other	28 (38.9)	17 (22.3)
Not defined	5 (6.9)	2 (2.6)
Client error	4 (5.5)	0
Pharmacy error	3 (4.1)	14 (18.4)
Prescribing error	0	0
Adverse reaction	0	0
Other	1 (1.3)	0
**Severity assessment classification (SAC) code (if reported)**		
1	0	0
2	0	0
3	6 (8.3)	2 (2.7)
4	66 (91.7)	71 (93.4)
WHO Anatomical Therapeutic Chemical (ATC) code *		
A—Alimentary tract and metabolism	9	7
B—Blood/blood forming organs	6	6
C—Cardiovascular	13	1
D—Dermatologicals	1	0
H—Systemic hormonal preparations	0	1
J—Anti-infectives for systematic use	2	1
M—Musculoskeletal system	2	2
N—Nervous system	16	15
R—Respiratory system	0	1
S—Sensory organs	1	0
**Other**		
DDA ^†^, not specified	18	35
Dose administration aid, not specified	0	2
Unknown/not recorded	5	1
**Medication administration route**		
Oral	41 (56.9)	41 (53.9)
Transdermal	21 (29.1)	32 (42.1)
Intramuscular	3 (4.1)	0
Subcutaneous	4 (5.5)	3 (3.9)
Topical	1 (1.3)	0
Rectal	1 (1.3)	0
Other	1 (1.3)	0

WHO: World Health Organization; * Three incidents involved more than one medication from more than one ATC code subgroup. ^†^ DDA, Drug of Dependence or Addiction. Schedule 8/Controlled drugs according to the Australian Poisons Standard, may include alprazolam, buprenorphine, codeine (except when included in schedule 4), dihydrocodeine (except when included in schedule 3 or 4), dihydromorphine, fentanyl, flunitrazepam, hydrocodone, hydromorphone, ketamine, methadone, methylphenidate, morphine, oxycodone, tapentadol.

**Table 3:** Medication incidents over the 12-month follow-up.

Months	Intervention Group (Incidents/100 Person-Years)	Comparison Group (Incidents/100 Person-Years)	Unadjusted Incidence Rate Ratio (95% CI ^†^)	Adjusted * Incidence Rate Ratio (95% CI ^†^)
0 to 4	118	59	1.99 (1.06–3.76)	1.40 (0.75–2.61)
5 to 8	79	58	1.37 (0.59–3.14)	1.09 (0.46–2.59)
9 to 12	58	85	0.69 (0.31–1.53)	0.58 (0.25–1.35)
0 to 12	95	66	1.44 (0.83–2.48)	1.13 (0.53–2.38)

* Adjusted for pre-rate, facility, region, gender and length of stay; ^†^ CI, confidence intervals.

## Data Availability

Participants of this study did not agree for their data to be shared publicly, so supporting data is not available.

## Supplementary Materials

The following are available online at https://www.mdpi.com/2077-0383/10/5/1104/s1, Table S1: Severity assessment code (SAC) matrix used to classify medication incidents at the participating residential aged care facilities, Table S2: Medications implicated in incidents by WHO Anatomical Therapeutic Chemical (ATC) code.

## References

[1] Aitken M., Gorokhovich L. Advancing the responsible use of medicines: Applying levers for change. SSRN Electron. J..

[2] World Health Organization (2017). Medication without Harm- Global Patient Safety Challenge on Medication Safety.

[3] Pharmaceutical Society of Australia PSA19: Minister Hunt Reaffirms Commitment to Addressing Medicine Safety. https://www.psa.org.au/psa19-minister-hunt-reaffirms-commitment-to-addressing-medicine-safety/.

[4] National Coordinating Council for Medication Error Reporting and Prevention about Medication Errors. https://www.nccmerp.org/about-medication-errors.

[5] Stowasser D.A., Allinson Y.M., O’Leary K.M. (2004). Understanding the medicines management pathway. J. Pharm. Pract. Res..

[6] Australian Pharmaceutical Advisory Council (2005). Guiding Principles to Achieve Continuity in Medication Management.

[7] Elliott R., Camacho E., Campbell F., Jankovic D., St James M.M., Kaltenthaler E., Wong R., Sculpher M., Faria R. Prevalence and economic burden of medication errors in the NHS in England. Rapid Evidence Synthesis and Economic Analysis of the Prevalence and Burden of Medication Error in the UK.

[8] Keers R.N., Williams S.D., Cooke J., Ashcroft D.M. (2013). Prevalence and nature of medication administration errors in health care settings: A systematic review of direct observational evidence. Ann. Pharmacother..

[9] Jokanovic N., Tan E.C., Dooley M.J., Kirkpatrick C.M., Bell J.S. (2015). Prevalence and factors associated with polypharmacy in long-term care facilities: A systematic review. J. Am. Med. Dir. Assoc..

[10] World Health Organization (2016). Medication Errors: Technical Series on Safer Primary Care.

[11] Tariq A., Georgiou A., Westbrook J. (2012). Medication incident reporting in residential aged care facilities: Limitations and risks to residents’ safety. BMC Geriatr..

[12] Roughead L., Semple S. (2013). Literature Review: Medication Safety in Acute Care in Australia. https://www.safetyandquality.gov.au/sites/default/files/migrated/Literature-Review-Medication-Safety-in-Australia-2013.pdf.

[13] Alldred D.P., Standage C., Fletcher O., Savage I., Carpenter J., Barber N., Raynor D.K. (2011). The influence of formulation and medicine delivery system on medication administration errors in care homes for older people. BMJ Qual. Saf..

[14] Aged Care Complaints Commissioner (2018). Annual Report 2017–2018.

[15] Ferrah N., Lovell J.J., Ibrahim J.E. (2017). Systematic review of the prevalence of medication errors resulting in hospitalization and death of nursing home residents. J. Am. Geriatr. Soc..

[16] Barber N.D., Alldred D.P., Raynor D.K., Dickinson R., Garfield S., Jesson B., Lim R., Savage I., Standage C., Buckle P. (2009). Care homes’ use of medicines study: Prevalence, causes and potential harm of medication errors in care homes for older people. Qual. Saf. Health Care.

[17] Szczepura A., Wild D., Nelson S. (2011). Medication administration errors for older people in long-term residential care. BMC Geriatr..

[18] Handler S.M., Perera S., Olshansky E.F., Studenski S.A., Nace D.A., Fridsma D.B., Hanlon J.T. (2007). Identifying modifiable barriers to medication error reporting in the nursing home setting. J. Am. Med. Dir. Assoc..

[19] Alldred D.P., Kennedy M.C., Hughes C., Chen T.F., Miller P. (2016). Interventions to optimise prescribing for older people in care homes. Cochrane Database Syst. Rev..

[20] Thiruchelvam K., Hasan S.S., Wong P.S., Kairuz T. (2017). Residential aged care medication review to improve the quality of medication use: A systematic review. J. Am. Med. Dir. Assoc..

[21] Gilmartin-Thomas J.F., Smith F., Wolfe R., Jani Y. (2017). A comparison of medication administration errors from original medication packaging and multi-compartment compliance aids in care homes: A prospective observational study. Int. J. Nurs. Stud..

[22] Baril C., Gascon V., St-Pierre L., Lagace D. (2014). Technology and medication errors: Impact in nursing homes. Int. J. Health Care Qual. Assur..

[23] McDerby N., Kosari S., Bail K., Shield A., Peterson G., Naunton M. (2019). The effect of a residential care pharmacist on medication administration practices in aged care: A controlled trial. J. Clin. Pharm. Ther..

[24] George J., Phun Y.T., Bailey M.J., Kong D.C.M., Stewart K. (2004). Development and validation of the medication regimen complexity index. Ann. Pharmacother..

[25] Alves-Conceição V., Silva D.T.D., Santana V.L., Dos Santos E.G., Santos L.M.C., de Lyra D.P. (2017). Evaluation of pharmacotherapy complexity in residents of long-term care facilities: A cross-sectional descriptive study. BMC Pharmacol. Toxicol..

[26] Lalic S., Sluggett J.K., Ilomaki J., Wimmer B.C., Tan E.C., Robson L., Emery T., Bell J.S. (2016). Polypharmacy and medication regimen complexity as risk factors for hospitalization among residents of long-term care facilities: A prospective cohort study. J. Am. Med. Dir. Assoc..

[27] Hwang Y., Yoon D., Ahn E.K., Hwang H., Park R.W. (2016). Provider risk factors for medication administration error alerts: Analyses of a large-scale closed-loop medication administration system using RFID and barcode. Pharmacoepidemiol. Drug Saf..

[28] Sluggett J.K., Chen E.Y.H., Ilomaki J., Corlis M., Hilmer S.N., Van Emden J., Ooi C.E., Nguyen K.H., Comans T., Hogan M. (2018). SImplification of Medications Prescribed to Long-tErm care Residents (SIMPLER): Study protocol for a cluster randomised controlled trial. Trials.

[29] Sluggett J.K., Chen E.Y.H., Ilomaki J., Corlis M., Van Emden J., Hogan M., Caporale T., Keen C., Hopkins R., Ooi C.E. (2020). Reducing the burden of complex medication regimens: SImplification of Medications Prescribed to Long-tErm care Residents (SIMPLER) cluster randomized controlled trial. J. Am. Med. Dir. Assoc..

[30] Sluggett J.K., Hopkins R.E., Chen E.Y.H., Ilomäki J., Corlis M., Van Emden J., Hogan M., Caporale T., Ooi C.E., Hilmer S.N. (2020). Impact of medication regimen simplification on medication administration times and health outcomes in residential aged care: 12 Month follow up of the SIMPLER randomized controlled trial. J. Clin. Med..

[31] Australian Institute of Health and Welfare GEN Aged Care Data. https://www.gen-agedcaredata.gov.au.

[32] Chen E.Y.H., Sluggett J.K., Ilomaki J., Hilmer S.N., Corlis M., Picton L.J., Dean L., Alderman C.P., Farinola N., Gailer J. (2018). Development and validation of the Medication Regimen Simplification Guide for Residential Aged CarE (MRS GRACE). Clin. Interv. Aging.

[33] SA Health Patient Incident Management Tool: Safety Assessment Code Matrix. https://sahealth.sa.gov.au.

[34] WHO Collaborating Centre for Drug Statistics Methodology (2020). Guidelines for ATC Classification and DDD Assignment.

[35] Charlson M.E., Pompei P., Ales K.L., MacKenzie C.R. (1987). A new method of classifying prognostic comorbidity in longitudinal studies: Development and validation. J. Chronic. Dis..

[36] Kaehr E., Visvanathan R., Malmstrom T.K., Morley J.E. (2015). Frailty in nursing homes: The FRAIL-NH scale. J. Am. Med. Dir. Assoc..

[37] Paradis E., Sutkin G. (2017). Beyond a good story: From Hawthorne Effect to reactivity in health professions education research. Med. Educ..

[38] Desai R.J., Williams C.E., Greene S.B., Pierson S., Caprio A.J., Hansen R.A. (2013). Exploratory evaluation of medication classes most commonly involved in nursing home errors. J. Am. Med. Dir. Assoc..

[39] Chen E.Y.H., Bell J.S., Ilomaki J., Keen C., Corlis M., Hogan M., Van Emden J., Hilmer S.N., Sluggett J.K. (2019). Medication regimen complexity in 8 Australian residential aged care facilities: Impact of age, length of stay, comorbidity, frailty, and dependence in activities of daily living. Clin. Interv. Aging.

[40] Lampert A., Seiberth J., Haefeli W.E., Seidling H.M. (2014). A systematic review of medication administration errors with transdermal patches. Expert Opin. Drug Saf..

[41] Chen E.Y.H., Bell J.S., Ilomaki J., Corlis M., Hogan M.E., Caporale T., Van Emden J., Westbrook J.I., Hilmer S.N., Sluggett J.K. Medication administration in Australian residential aged care: A time-and-motion study. J. Eval. Clin. Pract..

[42] Australian Government Department of Health Guiding Principles for Medication Management in Residential Aged Care Facilities. https://www.health.gov.au/resources/publications/guiding-principles-for-medication-management-in-residential-aged-care-facilities.

[43] Picton L., Lalic S., Ryan-Atwood T.E., Stewart K., Kirkpatrick C.M., Dooley M.J., Turner J.P., Bell J.S. (2020). The role of medication advisory committees in residential aged care services. Res. Soc. Admin. Pharm..

[44] Jheeta S., Franklin B.D. (2017). The impact of a hospital electronic prescribing and medication administration system on medication administration safety: An observational study. BMC Health Serv. Res..

[45] Elliott R.A., Lee C.Y., Hussainy S.Y. (2015). Evaluation of a hybrid paper-electronic medication management system at a residential aged care facility. Aust. Health Rev..

[46] Sluggett J.K., Ooi C.E., Gibson S., Angley M.T., Corlis M., E Hogan M., Caporale T., A Hughes G., Van Emden J., Bell J.S. (2020). Simplifying medication regimens for people receiving community-based home care services: Outcomes of a non-randomized pilot and feasibility study. Clin. Interv. Aging.

