# Supplementary material for: Impact of Medication Regimen Simplification on Medication Incidents in Residential Aged Care: SIMPLER Randomized Controlled Trial
Source: J Clin Med. 2021 Mar 6;10(5):1104. doi: 10.3390/jcm10051104 (PMC7961370; doi:10.3390/jcm10051104)
Supplement: Supplementary file 1 [file jcm-10-01104-s001.pdf]

Table S1. Severity assessment code (SAC) matrix used to classify medication incidents at the participating residential aged care facilities.

		Consequence				
		**Severe**	**Major**	**Moderate**	**Minor**	**Minimal**
Likelihood	**Frequent**	1	1	2	3	3
	**Likely**	1	1	2	3	3
	**Possible**	1	2	2	3	4
	**Unlikely**	1	2	3	4	4
	**Rare**	2	3	3	4	4

Table S2. Medications implicated in incidents by WHO† Anatomical Therapeutic Chemical (ATC) code.

Code	Drug group	Intervention group	Comparison group	Total cohort incidents
A	Alimentary tract, not spec.	0	1	1
A02B	Drugs for peptic ulcer/gastro-esophageal reflux disease	2	1	3
A06A	Drugs for constipation	1	2	3
A10A	Insulins and analogues	3	2	5
A10B	Blood glucose lowering, excluding insulin	1	0	1
A11	Vitamins, not spec.	1	0	1
A12A	Calcium supplements	1	1	2
B01A	Antithrombotic agents	6	5	11
B03B	Vitamin B_{12} and/or folic acid	0	1	1
C01D	Vasodilators, for cardiac disease	7	0	7
C02C	Anti-adrenergic agents, peripheral acting	2	0	2
C03C	High-ceiling diuretics	1	0	1
C03D	Potassium sparing diuretics	1	0	1
C07A	Beta-blocking agents	1	0	1
C08C	Selective calcium channel blockers	1	0	1
C09C	Angiotensin II receptor blockers	0	1	1
D	Dermatological, not spec.	1	0	1
H02A	Corticosteroids for systemic use	0	1	1
J01	Anti-bacterial for systemic use	2	1	3
M01A	Anti-inflammatory and anti-rheumatic	1	1	2
M03B	Centrally acting muscle relaxants	0	1	1
M04A	Anti-gout preparations	1	0	1
N02	Analgesics, not spec.	0	5	5
N02A	Opioids	3	0	3
N02B	Other analgesics or antipyretics	8	3	11
N03A	Anti-epileptics	1	0	1
N04	Anti-Parkinson's, not spec.	0	1	1
N04B	Dopaminergic agents	0	1	1
N05A	Antipsychotics	1	1	2
N05B	Anxiolytics	2	2	4
N06A	Antidepressants	1	1	2
N06D	Anti-dementia drugs	0	1	1
R06A	Antihistamines for systemic use	0	1	1
S01	Ophthalmological, not spec.	1	0	1

†WHO: World Health Organization
